# Colorectal Centre of Excellence (COCOE) Registry: A protocol for a prospective longitudinal cohort of children with Hirschsprung's disease and anorectal malformations in Canada

**DOI:** 10.1371/journal.pone.0356813

**Published:** 2026-08-28

**Authors:** Lee Hill, Étienne St-Louis, Mylène Dandavino, Nadine Korah, Gaël Kornitzer, Janie Benoit, Mohamed El-Sherbini, Courteney Allan, Chloé Vanier, Vanessa Smrk, Elena Guadagno, Dan Poenaru, Hussein Wissanji

**Affiliations:** 1 Harvey E. Beardmore Division of Pediatric Surgery, Montreal Children's Hospital, McGill University Health Centre (MUHC), Montréal, Quebec, Canada; 2 McGill University Faculty of Medicine and Health Sciences, Montreal, Quebec, Canada; 3 Department of Pediatrics, Division of General Pediatrics, The Montreal Children’s Hospital, McGill University Health Centre, Montreal, Quebec, Canada; 4 Department of Pediatrics, Division of Pediatric Gastroenterology and Nutrition, The Montreal Children’s Hospital, McGill University Health Centre, Montreal, Quebec, Canada; 5 Département d'obstétrique-gynécologie, Faculté de médecine, Université de Montréal, Montreal, Quebec, Canada; 6 Department of Pediatric Surgery, Division of Pediatric and Adolescent Gynecology, The Montreal Children’s Hospital, McGill University Health Centre, Montreal, Quebec, Canada; 7 Department of Pediatric Surgery, Division of Pediatric Urology, The Montreal Children’s Hospital, McGill University Health Centre, Montreal, Quebec, Canada; 8 Department of Physiotherapy, The Montreal Children’s Hospital, McGill University Health Centre, Montreal, Quebec, Canada; 9 School of Human Nutrition, McGill University, Montreal, Quebec, Canada; Macau University of Science and Technology, HONG KONG

## Abstract

Hirschsprung's disease (HD) and anorectal malformations (ARM) are congenital colorectal conditions requiring lifelong multidisciplinary care, yet no prospective, longitudinal registry exists for affected children in Canada. We describe the protocol for the COCOE Registry, the first prospective cohort of paediatric patients with HD and ARM at the Colorectal Centre of Excellence (COCOE), Montreal Children's Hospital, McGill University Health Centre, Montréal, Québec, Canada. The registry enrolls children aged 0–17 years with a documented diagnosis of HD or ARM on a continuous basis, with follow-up until age 18. Clinical data are abstracted from a standardised multidisciplinary assessment form and entered into REDCap. Patient-reported outcomes are collected using six validated instruments spanning disease-specific quality of life, continence, general well-being, family functioning, parental adjustment, and bowel management experience. Statistical analyses will include descriptive summaries, group comparisons, survival analyses, and multivariable logistic regression to identify factors associated with key outcomes, reported in accordance with STROBE guidelines. Research ethics approval has been granted by the McGill University Health Centre Research Ethics Board (MUHC REB No. 2026–10997). This study is registered with ClinicalTrials.gov (NCT07603232). The COCOE Registry will generate longitudinal data to improve clinical follow-up, inform healthcare planning, support equitable transitions to adult care, and enable participation in international multicentre research.

## Introduction

Colorectal disorders, particularly Hirschsprung's disease (HD) and anorectal malformations (ARM), represent significant challenges in paediatric healthcare, with lifelong implications for affected children and their families [1–4]. HD is a congenital condition characterised by the absence of ganglion cells in the distal colon, leading to functional intestinal obstruction [1]. In Ontario, Canada, the incidence of HD has been estimated at 2.05 per 10,000 live births [1]. ARM, which encompasses a spectrum of congenital anomalies affecting the anorectal region and frequently associated organ systems, occurs at approximately 1 in 2,162 births in Alberta, with a notable male prevalence (1.7:1) [3,4]. Both conditions require early, often complex, surgical intervention and are associated with lifelong impacts on quality of life, growth, and development.

The management of paediatric colorectal disorders has advanced significantly with the development of specialised multidisciplinary centres, such as the Montreal Children's Hospital's Colorectal Centre of Excellence (COCOE).^8^ This centre is the first of its kind in Québec. Despite advances, children with these disorders often face ongoing challenges, including multiple surgeries, bowel management programmes, and psycho-social support needs. Long-term follow-up is critical, as many patients experience complications such as faecal incontinence, enterocolitis, and issues related to transition from paediatric to adult care [5–7]. For families, these challenges translate into substantial emotional and financial strain, particularly as patients age out of paediatric services [8–10].

Internationally, the importance of systematic data collection has been recognised through the establishment of prospective registries and research consortia. The European Anorectal Malformation Network (ARM-Net) [11,12] and the Paediatric Colorectal and Pelvic Learning Consortium (PCPLC) [13] are leading examples. The PCPLC registry has enabled robust, multicentre research: Rice-Townsend et al [14] found that only 24% of patients with ARM achieved full bowel control across over 500 patients, and Kastenberg et al. [15] demonstrated that rates of postoperative enterocolitis remain high regardless of surgical timing.

Canada has yet to establish a prospective paediatric colorectal registry. Such a registry would allow tracking of long-term outcomes and quality of life, inform continuity of care across the paediatric-to-adult transition, support healthcare planning and resource allocation, and enable participation in international collaborative research [6,11]. We therefore propose the COCOE Registry: a prospective, longitudinal registry of paediatric patients with colorectal disease attending the Montreal Children's Hospital.

## Objectives

The objectives of the COCOE Registry are: 1) to establish the first prospective registry of paediatric patients with HD and ARM in Canada; 2) to collect and analyse longitudinal data on incidence, clinical outcomes, and treatments; 3) to collect patient-reported outcomes on well-being, quality of life, and familial stress and functioning; 4) to improve understanding of regional variations in care and outcomes; and 5) to identify factors contributing to loss to follow-up.

## Methods and analysis

### Study design

**Fig 1:**
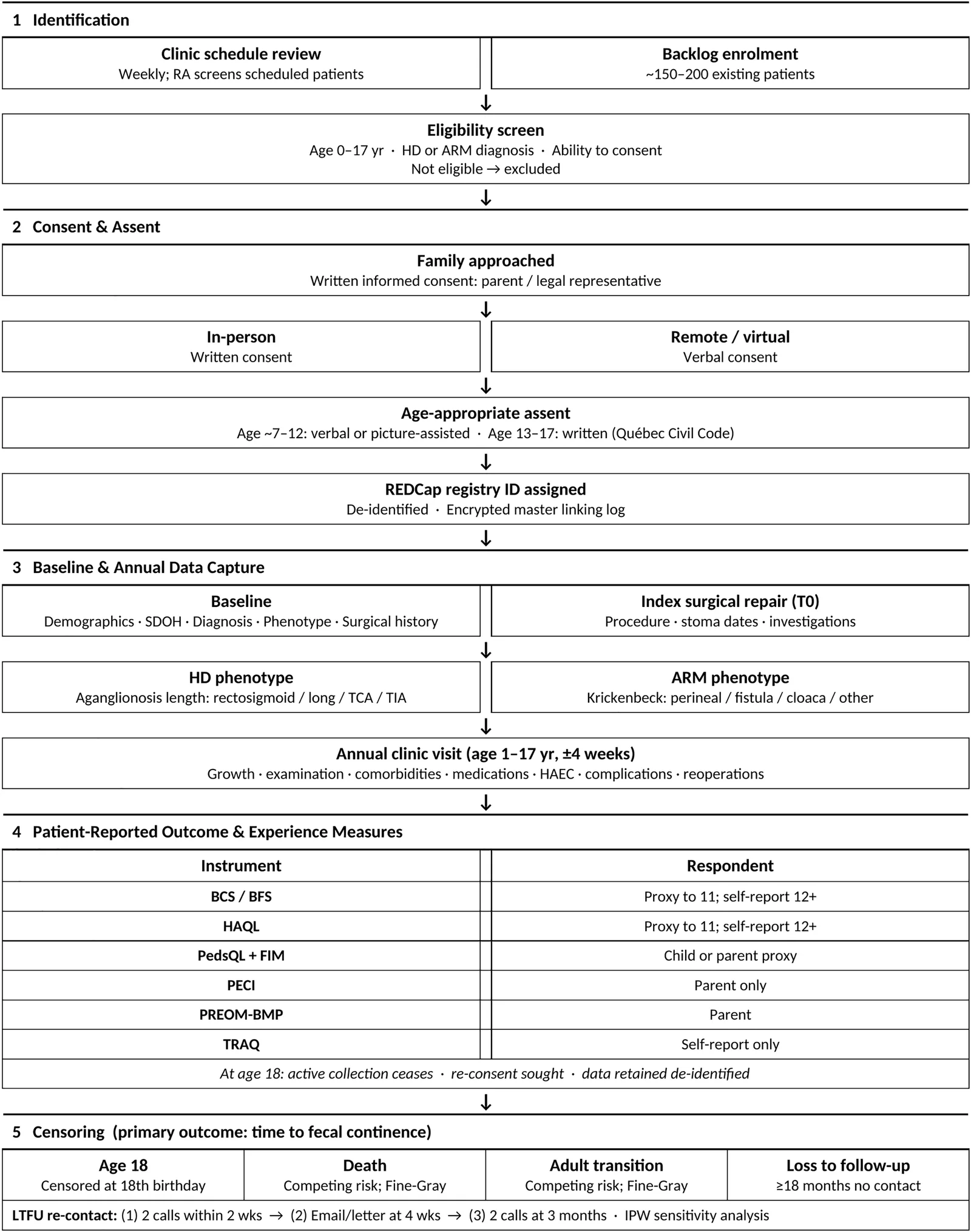
COCOE Registry workflow. Identification of eligible patients, consent and assent, baseline and annual data capture, patient-reported outcome administration schedule, and censoring definitions.

This study follows a longitudinal, prospective observational design. The registry has no anticipated completion date; patients are recruited on a continuous basis and followed until they reach 18 years of age. Fig 1 summarises the registry workflow, from identification of eligible patients through consent and baseline data capture to the schedule of longitudinal follow-up assessments and the definitions applied at censoring.

### Study population and setting

Eligible paediatric patients (aged 0–17 years) with documented diagnoses of HD or ARM are invited to participate at the COCOE clinic, Montreal Children's Hospital. Prospective patients are identified through weekly reviews of colorectal clinic schedules.

Although HD and ARM are embryologically distinct conditions with different surgical approaches and long-term outcome profiles, both require lifelong multidisciplinary follow-up and are managed by the same specialist team at COCOE. Enrolling both conditions within a single registry infrastructure reflects this shared clinical pathway and the impracticality of maintaining two separate registries at a single centre. To respect these clinical and methodological distinctions, primary and multivariable analyses will be conducted separately for each disease cohort rather than pooled, with disease-specific instruments and predictors as detailed in the Statistical Analysis section.

### Catchment and representativeness

The Montreal Children’s Hospital is one of two paediatric tertiary care centres in Québec and receives colorectal referrals from across the province, including from Nunavik and the Cree territory of James Bay, as well as selected complex referrals from other provinces. The registry therefore captures a substantial proportion, but not the entirety, of the incident paediatric HD and ARM population in Québec. To quantify representativeness rather than assert it, the age, sex, diagnosis and severity distribution of enrolled participants will be compared at each annual analysis against two external benchmarks: (i) expected provincial case counts, derived by applying published Canadian incidence estimates for HD (2.05 per 10,000 live births [1]) and ARM (approximately 1 in 2,162 births [3,4]) to Québec live-birth denominators; and (ii) the published case-mix of the ARM-Net and PCPLC registries [11,13]. Aggregate, non-identifying information on patients who are approached but decline enrollment (age band, diagnosis, and referral region) will also be recorded so that a non-participation comparison can be reported. Any material divergence, for example under-representation of remote communities or of the most severe Krickenbeck categories, will be reported explicitly alongside registry outputs and taken into account when interpreting external validity.

### Eligibility criteria

Inclusion and Exclusion Criteria are described in Table 1.

**Table 1:** Inclusion and exclusion criteria.

Inclusion criteria	Exclusion criteria
Aged 0–17 years	Aged 18 years or older
Documented diagnosis of HD or ARM	No diagnosis of HD or ARM
Patients and/or their caregivers or authorised representatives must be able to provide informed consent	Unable to provide consent

### Outcome measures

The primary outcome and its definition are described in detail below. Pre-specified secondary outcomes are presented in Table 2 below, each with its definition, instrument or data source, timing of ascertainment, and analytic role.

**Table 2:** Pre-specified primary and secondary outcomes.

Outcome	Definition	Instrument / Data Source	Timing of Ascertainment	Priority	Analytic Role
**Time to fecal continence**	First clinic visit (confirmed at subsequent visit) at which the participant meets social continence threshold: BCS criteria (ARM) or BFS ≥ 17 (HD), from date of definitive repair	BCS (ARM) [16] or BFS (HD) [17], parent/caregiver proxy to age 11; self-report from age 12	Annual visits from age 3	**PRIMARY**	Kaplan–Meier estimation; Cox proportional hazards regression; Fine-Gray model for competing risks; landmark analyses at ages 5 and 10
HD-associated enterocolitis (HAEC)	≥1 physician-confirmed episode meeting Ruttenstock criteria requiring medical treatment	Clinical record review; REDCap adverse event log	Each annual visit; first episode date recorded	Secondary 1	Cox regression / Fine-Gray competing risks; descriptive incidence rate
Reoperation rate	Any unplanned surgical return to the operating room following index repair	Operative report review; REDCap surgical procedure log	Cumulative from index repair; each annual visit	Secondary 2	Cox regression / Fine-Gray competing risks; cumulative incidence curve
Disease-specific quality of life	HAQL total score and subscale scores (age-appropriate version)	HAQL questionnaire, self- or proxy-report [18]	Ages 6, 10, and 14 years	Secondary 3	Linear mixed-effects model (repeated measures); descriptive means by group
Transition readiness	Transition Readiness Assessment Questionnaire	TRAQ [19], self-report	Ages 14 and 17 years	Secondary 4	Linear mixed-effects model; descriptive score trajectory
Loss to follow-up (LTFU)	No clinic encounter, survey, or successful contact for ≥18 months while under age 18 and not yet transitioned	REDCap contact log; clinic attendance record	Flagged at 18-month mark; reviewed at each annual analysis	Secondary 5	Logistic regression (ever-lost vs retained); descriptive LTFU rate

The primary outcome is time to achievement of fecal continence from the date of definitive surgical repair, analysed as a time-to-event outcome. For participants with ARM, continence is assessed using the Baylor Continence Scale (BCS) [16], with social continence defined as no soiling episodes in the preceding 4 weeks per published BCS criteria, completed by the parent or caregiver proxy up to age 11 and by self-report from age 12. For participants with HD, continence is assessed using the Bowel Function Score (BFS) [17], with social continence defined as a BFS score ≥ 17, administered to the parent or caregiver proxy up to age 11 and by self-report from age 12. Both instruments are administered at every annual clinic visit beginning at age 3 years. The earliest clinically meaningful assessment for continence is age 3, consistent with normal developmental milestones for voluntary bowel control. Participants will be censored at age 18, death, transition to adult care, or loss to follow-up, whichever occurs first.

Pre-specified secondary outcomes, listed in priority order, are: (1) Hirschsprung-associated enterocolitis (HAEC), defined as at least one physician-confirmed episode meeting the Ruttenstock diagnostic criteria and requiring medical treatment, assessed at each annual visit [20]; (2) reoperation rate, defined as any unplanned surgical return to the operating room following index repair, assessed cumulatively; (3) disease-specific quality of life, measured using the HAQL [18] at ages 6, 10, and 14 years using age-appropriate versions; (4) transition readiness, assessed at ages 14 and 17 years using the validated Transition Readiness Assessment Questionnaire (TRAQ) [19], and (5) loss to follow-up rate, defined operationally in the Loss to Follow-Up section. Each secondary outcome will be reported descriptively and, where the events-per-variable threshold is met (see Sample Size section), included as an endpoint in pre-specified multivariable analyses with the analytic role specified in the Statistical Analysis section.

### Sample size and expected enrolment

The registry is designed for indefinite, continuous enrollment. We distinguish two enrollment streams: incident cases (children newly diagnosed with HD or ARM presenting to COCOE, estimated at a minimum of 5 per month) and previous cases (patients previously diagnosed but lost to follow-up and re-engaged, estimated at approximately 3 per month). Combined, the registry aims to enroll approximately 96 participants per year. Based on a prevalent backlog of an estimated 150–200 patients currently followed at COCOE, total enrollment over 5 years is projected at 480–680 participants.

While no minimum sample size is required for registry operation, we provide a feasibility projection. Based on published ARM cohort data reporting approximately 24–30% achieving full fecal continence [14], and HD cohort data reporting approximately 39% achieving a BFS ≥ 17 by adolescence, we project a combined continence event rate of approximately 30–35% within the first 5 years of registry operation [21]. This corresponds to approximately 145–240 continence events across the projected 480–680 enrolees, supporting inclusion of up to 14–24 covariates in the primary Cox model at an events-per-variable (EPV) threshold of 10 [22]. Importantly, this time-to-event framing includes all enrolled participants regardless of age at enrollment, resolving the limitation of the age-5 cross-sectional design by allowing older prevalent cases to contribute person-time and events from their enrolment date. Pre-specified multivariable analyses will be triggered only once this EPV threshold is confirmed at interim review; otherwise analyses will be limited to descriptive and unadjusted comparisons.

### Study timeline and milestones

The registry opened to enrollment on 3 ^rd^ November 2025 and enrollment is ongoing. Formal analyses have yet to be conducted at the time of publication. Below is outlined the Projected Milestones and Study Timeline.

Projected milestones and Study Timeline are as follows:

**Year 1:** incident enrollment fully operational and backlog enrollment at least 25% complete;

**Year 2:** first annual data quality audit completed and first descriptive summary manuscript submitted;

**Year 3:** first interim analysis of the primary outcome conducted for participants who have reached age 5, if the EPV threshold is met;

**Year 4 and 5:** comprehensive registry report including all pre-specified multivariable analyses submitted for publication.

Milestone dates will be updated in subsequent protocol versions as the registry matures. As the study is a longitudinal prospective study, intermittent analyses of the data will be conducted throughout the study duration as is outlined in the Projected Milestones above.

### Participant recruitment and consent

Written informed consent is obtained from parents or legally authorised representatives at enrollment. Age-appropriate assent is sought from all participants with developing decision-making capacity, consistent with TCPS2 Article 4.2 [23] and MUHC REB guidelines: verbal or picture-assisted assent is used for children aged approximately 7–12 years, and written assent is obtained from adolescents aged 13–17 years, consistent with the age of capacity for research participation under the Québec Civil Code [24]. Assent is documented in REDCap alongside the consent record.

Verbal consent is obtained where written consent is not feasible (e.g., telephone or virtual visits, or when translation is required) and is documented [23], with audio recording where permitted. Participation is entirely voluntary and has no bearing on the care received at Montreal Children's Hospital.

All registry data are de-identified: each participant is assigned a unique registry identifier, and names do not appear on any data collection forms. A master linking log is maintained separately on a password-protected encrypted drive accessible only to the PI. Data are not anonymised in the irreversible sense,  re-linkage to the participant remains possible during active follow-up for clinical and re-contact purposes. Full anonymisation, meaning permanent removal of all re-identification keys, will be applied only upon permanent withdrawal or at study closure.

When a participant turns 18 years of age, active data collection ceases. Data already collected will be retained in de-identified form pursuant to Section 12 of the Act Respecting the Protection of Personal Information in the Private Sector (Québec Law 25, as amended by Bill 64 [25]), which permits continued use of de-identified data for research purposes without renewed consent, subject to ongoing REB oversight (MUHC REB No. 2026–10997).

### Loss to Follow-Up

A participant will be considered lost to follow-up if there has been no clinic encounter, completed survey, or successful contact (by telephone, email, or mail) for 18 or more consecutive months in a participant aged under 18 years who has not yet transitioned to adult care or withdrawn from the registry.

When a participant misses a scheduled visit without prior notification, the research assistant will initiate a standardised re-contact sequence: (1) two phone call attempts within 2 weeks; (2) email or written letter within 4 weeks, if contact information is on file; and (3) two further phone call attempts at 3 months. If no successful contact is made after this full sequence, the participant will be flagged as lost to follow-up in REDCap with the date of last confirmed contact recorded. All contact attempts and outcomes will be logged prospectively.

Rates and timing will be reported descriptively at each annual analysis. In survival and longitudinal analyses, the loss will be treated as censoring at the date of last confirmed contact. The potential impact of informative (non-random) censoring will be examined using inverse probability weighting (IPW) as a sensitivity analysis [26,27]. Factors independently associated with the loss will be examined using logistic regression (ever-lost vs retained) as a secondary analysis, with candidate predictors pre-specified to include: diagnosis type, age at enrollment, sex, aganglionosis length or ARM Krickenbeck category, area-level deprivation quintile, and distance from COCOE.

## Data collection

### Clinical variables

Data collection is based on the standardised COCOE multidisciplinary clinical assessment form, adapted for input into REDCap [28,29]. This form captures all relevant clinical information encountered during routine care, including: patient demographics; referral details; primary diagnosis; involvement of subspecialists; current medications and allergies; vaccination status; detailed birth and surgical history; prior hospitalisations; relevant investigations; and multidisciplinary assessments. It also documents gastrointestinal, urological, gynaecological, dietary, developmental, social, and family history, as well as physical examination findings, growth parameters, and care plans. For research purposes, only de-identified data elements required to address the study objectives are abstracted, ensuring no additional procedures or burden beyond routine clinical care.

### Data abstraction procedures and reliability

To ensure consistency of abstraction from the multidisciplinary assessment form, the registry operates under a written data dictionary and standard operating procedure that specify, for every REDCap field, the permitted values, the source document of record, and the rule to be applied when sources disagree. Operative reports and histopathology reports take precedence over clinic correspondence for surgical and diagnostic variables; the multidisciplinary assessment form takes precedence for functional and psycho-social variables. All abstractors complete a standardised training package and must reach at least 90% field-level agreement with the Study Coordinator across five benchmark charts before abstracting independently. REDCap instruments use closed-response fields, branching logic, and range and cross-field validation rules wherever possible, so that free-text entry is reserved for variables that cannot be pre-coded [28,29].

A random 10% sample of records is independently re-abstracted each quarter by a second trained abstractor blinded to the original entry. Agreement will be quantified using Cohen’s kappa for categorical variables and the intra-class correlation coefficient for continuous variables, against a pre-specified acceptability threshold of κ ≥ 0.80 or ICC ≥ 0.80. Fields falling below this threshold will trigger review of the field definition, retraining of abstractors, and re-abstraction of affected records. Reliability statistics will be reported in the first descriptive registry manuscript and at each subsequent annual audit.

Adherence to the protocol is itself treated as a monitored registry metric. The proportion of eligible patients approached, the proportion consented, the proportion of scheduled assessments completed within their pre-specified age window, and instrument-level completion rates are reported quarterly to the registry Director. Pre-specified action thresholds, for example an assessment-window adherence rate below 70% across two consecutive quarters, trigger review of clinic workflow and, where necessary, a protocol amendment. These adherence metrics will be published alongside outcome data so that readers can judge the completeness and fidelity of the underlying datasets.

Variables collected will include: demographic information (parent and child age, sex); Social Determinants of Health (gender, race/ethnicity, income, education, and first three digits of the postal code-based Material and Social Deprivation Index); health services utilisation (emergency room/outpatient visits and inpatient admissions over the past year); clinical data (diagnosis with severity, surgical procedures with dates, prematurity, comorbidities, and complications); and Patient-Reported Outcome and Experience Measure (PROM/PREM) data.

### Social determinants of health

(a) Individual-level variables, collected directly from participants and caregivers using Statistics Canada standard wording [30]: sex assigned at birth (male, female, intersex); gender identity (man/boy, woman/girl, non-binary, another gender identity, prefer not to answer); race/ethnicity (self-identified, using Statistics Canada visible minority and Indigenous identity categories); household income before taxes (six categories: < $20,000; $20,000–$39,999; $40,000–$59,999; $60,000–$79,999; $80,000–$99,999; ≥ $100,000, aligned with Low Income Measure thresholds for Québec); highest level of education completed by the primary caregiver (less than high school diploma; high school diploma or equivalent; post-secondary certificate or diploma; bachelor's degree; graduate or professional degree); and employment status of the primary caregiver. For all individual-level SDOH variables, a “prefer not to answer” option will be provided and treated as missing in analyses, with missingness described and addressed using the plan detailed in the Statistical Analysis section.
(b) Area-level deprivation, derived from the Pampalon Material and Social Deprivation Index (MSDI), 2021 census-based [31], linked to participant residence via the first three characters of the postal code (forward sortation area, FSA) using the Statistics Canada Postal Code Conversion File. Material deprivation (a composite of employment, income, and education) and social deprivation (a composite of living alone, separated/widowed/divorced status, and lone-parent status) will be treated as separate dimensions and analysed as quintile scores (Q1 = least deprived to Q5 = most deprived). [32] The FSA will not appear in any publication output.

### Severity and phenotype

For ARM, anatomical and surgical severity will be classified using the Krickenbeck International Classification [33], comprising the following major clinical groups: perineal fistula; rectourethral fistula (bulbar or prostatic); rectovesical fistula; vestibular fistula; cloaca; no fistula; and rectal atresia/stenosis. For HD, severity will be characterised by length of aganglionosis confirmed by operative report and histopathology, using the following categories: short-segment (rectosigmoid transition zone); long-segment (transition zone beyond the sigmoid colon); total colonic aganglionosis (TCA); and total intestinal aganglionosis (TIA) [34]. Separate structured fields will capture: syndromic status (yes/no; if yes, syndrome name including trisomy 21, VACTERL syndrome association, RET proto-oncogene variants, and other named syndromes); and associated anomalies, documented using a structured checklist covering vertebral, cardiac, tracheo-oesophageal, renal, and limb anomalies (VACTERL) [35], with additional free-text fields for genitourinary, spinal, and other anomalies outside this classification.

### Patient-reported outcomes

During clinic visits or between encounters (online or paper), families are asked to complete validated surveys addressing multiple domains of well-being (Table 3). Surveys are available in English and French. Instruments include the Hirschsprung's and Anorectal Malformation Quality of Life (HAQL) questionnaire (disease-specific symptoms and quality of life, with age-specific and stoma-specific versions) [18]; the Baylor Continence Scale (BCS) [16], evaluating the social and functional impact of continence issues; the PedsQL General Well-being Scale [36]; the PedsQL Family Impact Module [36]; the Parent Experience of Child Illness (PECI) [37]; and, for those in bowel management programmes, the Patient-Reported Experience and Outcome Measures in Bowel Management Programmes (PREOM-BMP) [38]. Both French and English validated versions are available and will be used according to family language preference. Proxy-report by the parent or caregiver will be used for children aged 6–11 years; self-report will be used from age 12 onward, consistent with the validated age thresholds for each version.

**Table 3:** Summary of patient-reported outcome surveys.

Domain	Scale	Duration	Respondent	Brief description
Disease-specific	HAQL and BFS	10–20 min	Child (older) or Parent	Multiple-choice questions about symptoms and QoL; age-specific versions; stoma-specific items.
Social impact	BCS	10–15 min	Child (older) or Parent	23 questions on social continence and impact on life; self- or proxy-report.
General well-being	PedsQL General Well-being	4–10 min	Child or Parent (proxy)	Flexible item banks and short forms assessing physical, mental, and social health; domains include pain, fatigue, physical function, depression, anxiety, and social function.
Family functioning	PedsQL FIM	10–15 min	Parent	36-item parent-report scale assessing physical, emotional, social, and cognitive functioning, communication, worry, daily activities, and family relationships.
Parental well-being	PECI	10–15 min	Parent	25-item measure of adjustment to caring for a child with chronic illness; covers guilt/worry, unresolved sorrow/anger, long-term uncertainty, and emotional resources.
Family experience	PREOM-BMP	10–15 min	Parent	33-item survey for families of children in bowel management programmes; covers physical/emotional health, QoL, treatment success, financial considerations, schooling, and social concerns.

*BCS, Baylor Continence Scale; FIM, Family Impact Module; HAQL, Hirschsprung's and Anorectal Malformation Quality of Life; PECI, Parent Experience of Child Illness; PREOM-BMP, Patient-Reported Experience and Outcome Measures in Bowel Management Programmes; QoL, quality of life.*

For the purposes of this registry, a bowel management programme is defined as a structured, clinician-supervised regimen of enemas, laxatives, or dietary modification,  prescribed and monitored by the COCOE team,  for the management of fecal incontinence or constipation that has not responded to first-line measures. Eligibility for the PREOM-BMP is determined at each clinic visit by the responsible clinician and recorded in REDCap. Based on current COCOE caseload, approximately 40–50% of enrolled participants are expected to be in a formal bowel management programme at any given assessment visit.

### Survey administration and burden mitigation

The six instruments have a combined estimated completion time of 54–95 minutes depending on age and programme status. To reduce burden, instruments are administered in a staggered fashion across visits: the HAQL, BCS and BFS are prioritised at each visit; the PedsQL modules and PECI are administered at alternating annual visits; and the PREOM-BMP is administered only to eligible families. Where possible, surveys are distributed electronically via REDCap prior to the clinic visit to allow completion at home, reducing in-clinic time. Paper versions are available for families without internet access, and a trained research assistant is available to assist with completion where literacy or language is a barrier. Surveys are available in English and French. In the event of partial completion, available items will be scored according to the instrument's published scoring guidelines; instruments with fewer than the minimum required items completed will be recorded as missing and addressed per the missing data plan in the Statistical Analysis section. Based on experience from comparable paediatric surgical registries, we anticipate an instrument completion rate of approximately 70–80% per visit; completion rates will be reported at each annual analysis.

## Data management

Data are collected at each clinic visit by study team members with approved access to the medical record. Online surveys are created and collected directly in REDCap; paper surveys and telephone surveys are entered by a team member. REDCap (Research Electronic Data Capture) is a secure, web-based platform hosted at the Research Institute of the McGill University Health Centre (RI-MUHC) that provides validated data capture, audit trails, automated export procedures, and data interoperability [28,29].

## Statistical analysis

Analyses are pre-specified by objective as follows.

**Objective 1:** Incidence and clinical outcomes. Descriptive summaries of diagnosis, surgical procedures, and complication rates will be reported using means (SD) or medians (IQR) for continuous variables and frequencies (%) for categorical variables [39]. Time-to-event outcomes (e.g., first HAEC episode, reoperation) will be analysed using Cox proportional hazards regression. Where competing risks are present (e.g., death or transition to adult care prior to the event of interest), the Fine-Gray subdistribution hazard model will be applied [40]. Candidate predictors for each time-to-event model are pre-specified as: diagnosis type, Krickenbeck category or aganglionosis length, age at index repair, sex, syndromic status, and area-level deprivation quintile.

**Objective 2:** Patient-reported outcomes. Longitudinal PROM/PREM data collected across multiple clinic visits will be analysed using linear mixed-effects models (for continuous scores) or generalised estimating equations (GEE; for binary or count outcomes), with an unstructured or autoregressive covariance structure selected by the Akaike Information Criterion [41]. Cross-sectional group comparisons at a single timepoint will use chi-square or Fisher's exact tests (categorical) and independent-samples t-tests or Mann-Whitney U tests (continuous), as appropriate.

**Objective 3:** Factors associated with key outcomes. Time to achievement of fecal continence will be estimated using the Kaplan–Meier method, with curves reported separately for ARM and HD given the use of disease-specific instruments. Given the embryological, surgical, and prognostic distinctions between the two conditions, and the use of disease-specific continence instruments, multivariable analyses will be conducted separately for ARM and HD rather than as a single combined model. For each disease cohort, a Cox proportional hazards model will be fitted with disease-appropriate pre-specified candidate predictors: for ARM, Krickenbeck category, age at index repair, sex, syndromic status, and area-level deprivation quintile; for HD, length of aganglionosis, age at index repair, sex, syndromic status, and area-level deprivation quintile. The proportional hazards assumption will be tested for each model using Schoenfeld residuals. Where competing risks are present (death or transition prior to the continence event), the Fine-Gray subdistribution hazard model will be applied within each cohort. Pre-specified landmark analyses will report the proportions achieving continence by age 5 and age 10 for each disease, to allow benchmarking against published ARM and HD cohort data. For each disease-specific model, multivariable Cox regression will be triggered only once the events-per-variable (EPV) threshold of 10 is met within that cohort at interim review; otherwise analyses for that cohort will be limited to descriptive and unadjusted comparisons.

### Missing data

Missing data will be characterised in terms of amount and pattern at each planned analysis. For multivariable analyses, data missing at random (MAR) will be handled using multiple imputation by chained equations (MICE) with 20 imputed datasets pooled using Rubin's rules [42]. Complete-case analyses will serve as the primary approach and MICE results as sensitivity analyses. Where data missing not at random (MNAR) cannot be excluded (particularly for SDOH variables where “prefer not to answer” may be non-random), best-case and worst-case sensitivity analyses will be conducted [42].

Missing patient-reported outcome data and incomplete follow-up are anticipated to be the principal analytic challenges over the observation window to age 18, and are addressed by a pre-specified hierarchy of methods. At the item level, partially completed questionnaires will be prorated according to the developer’s published scoring rule, and scale scores will be set to missing where fewer than the minimum required items are available. At the visit level, longitudinal analyses of PRO scores will use linear mixed-effects models and generalised estimating equations, which retain participants with incomplete visit series and yield valid inference under a missing-at-random assumption [41]. For multivariable models requiring complete covariate vectors, multiple imputation by chained equations will be used, with the imputation model including the analysis model variables, the outcome, and auxiliary variables predictive of non-response (age band, distance from COCOE, deprivation quintile, language of administration, and number of previously missed appointments) [42].

As loss to follow-up and PRO non-response may both be informative, inverse probability of censoring weights estimated from these same predictors will be applied as a sensitivity analysis to the survival and longitudinal models [26,27]. Robustness to departures from the missing-at-random assumption will be assessed using pattern-mixture models in which imputed PRO scores are delta-adjusted by 0.2, 0.5, and 0.8 standard deviations in the unfavourable direction. The amount and pattern of missingness, by instrument, visit, and disease cohort, will be tabulated in every registry publication, and no outcome analysis will be reported without the accompanying completeness figures. A pre-specified threshold of more than 40% missingness for a given instrument at a given assessment point will preclude model-based inference for that instrument at that point, which will instead be reported descriptively.

This protocol paper has additionally been prepared with reference to the SPIRIT 2025 statement (Standard Protocol Items: Recommendations for Interventional Trials) [43], adapted for observational registry protocols. A completed SPIRIT checklist is provided as S1 File.

## Monitoring and data quality oversight

Given the observational, registry-based design of this study, a formal independent data monitoring committee is not required. Data quality will be overseen by the registry Director (HW) and the Study Coordinator. Routine data quality checks will be performed quarterly, including review of completeness, range checks, and cross-field consistency within REDCap. Any data queries will be resolved by the data abstractors in consultation with the clinical team. An annual internal audit of a random sample of records against source documents will be conducted to assess data accuracy. Audit findings, inter-abstractor reliability statistics, and the protocol adherence metrics described above will be summarised in an annual data quality report, reviewed by the COCOE steering group and retained for reporting to the MUHC REB.

## Protocol amendments

Any substantive amendments to this protocol, including changes to eligibility criteria, data collection instruments, or consent procedures, will be submitted to the MUHC REB for approval prior to implementation. Non-substantive administrative amendments will be documented in a protocol amendment log maintained by the Study Coordinator. All approved amendments will be reflected in an updated protocol version with a revised version number and date, and will be reported in any resulting publications.

## Ethnicity and cultural safety

This research may involve participants from First Nations, Inuit, and Métis communities. Following consultation with an Indigenous research advisor, a formal Cultural Safety Review was determined not required. The consultation concluded that the study design does not involve data collection specific to or within Indigenous communities, and that the scope and conduct of the registry do not meet the threshold requiring a formal Cultural Safety Review under current institutional guidelines. Ethnicity data will be collected solely for demographic description, using Statistics Canada Indigenous identity categories, with participants having the option to self-identify broadly as Indigenous, First Nations, Métis, or Inuit. No population-specific sub-analyses will be conducted and no identifiers linking participants to specific communities will appear in any analysis or publication output. The study team acknowledges that the area-level Pampalon MSDI, derived at the forward sortation area level, may not adequately capture the social and material circumstances of Indigenous participants in urban Montréal due to within-FSA heterogeneity; this is noted as a study limitation. Future iterations of the registry will consider engagement with urban Indigenous health organisations to inform culturally appropriate data collection.

## Patient and public involvement

To ensure patient-centred research, a Patient Partner Committee is being established with support from Unité de soutien SSA-Québec – RUISSS McGill. Patient partners will actively shape research priorities, refine study designs, interpret results, provide guidance on ethical considerations and knowledge translation strategies, and continuously disseminate findings within patient and social networks. This study follows the Strategy for Patient-Oriented Research (SPOR) patient engagement principles [44].

## Ethics and dissemination

### Ethics approval

This study is conducted in accordance with the Tri-Council Policy Statement: Ethical Conduct for Research Involving Humans (TCPS2) [23]. Ethics approval has been granted by the McGill University Health Centre Research Ethics Board (MUHC REB No. 2026–10997), which is responsible for monitoring the study at all participating institutions in the health and social services network in Québec. Authorisation to access patient charts is obtained from the Director of Professional Services (DPS). Access occurs only after consent has been obtained from the patient and family.

### Dissemination policy

Findings will be disseminated regardless of direction or magnitude of results.

### Confidentiality and data governance

All data collected from a participant are considered personal information and are kept confidential within the limits of the law. Upon enrollment, each participant is assigned a unique registry identifier known only to the principal investigator, co-investigators, and research assistants. Participant names do not appear on any data collection forms. Data within the registry are de-identified. The only protected health information collected includes dates (birth, discharge, death); for publication, dates are converted to relative units (e.g., days from birth), birth year is provided, and no centre identifiers are released.

The data registry is situated within and under the auspices of the MUHC Department of Paediatric Surgery. Electronic data are stored on password-protected computers in administrative offices. Paper records and signed consent forms are stored in a locked filing cabinet in a locked room. The REDCap server is located within the IT department of the Research Institute of the MUHC. Access to the registry is restricted to authorised personnel (the principal investigator (PI), co-principal investigators, and students/trainees granted explicit access). The master linking log is stored on a password-protected encrypted external USB drive kept in a locked cabinet in the principal investigator's office.

Participants may withdraw from the registry at any time by verbal or written request. Upon withdrawal, clinical data are destroyed and personal information deleted from paper and computer records. A confirmation letter is provided. Research results already obtained from analysis of registry data will not be destroyed and may be used for other research projects (subject to ethical approval).

The guardian of the data is the principal investigator, Dr. Hussein Wissanji. If Dr. Wissanji leaves the Montreal Children's Hospital, guardianship is transferred to the new principal investigator. The data will be stored for as long as the registry has scientific interest and the Director can ensure its management.

### Potential benefits and harms

There are no direct benefits to patients or caregivers from participating in this registry; however, participants may derive satisfaction from contributing to a national dataset that will improve understanding of colorectal disease management and outcomes. There are no known harms. The potential for privacy breach is minimised by strict confidentiality and de-identification protocols. Participants may have access to their research file to verify and correct their data. Third parties such as insurers or employers may not access registry data unless authorised by a court of law or by the person who contributed the data. Participants will not incur additional costs and will not receive compensation, though a certificate of participation is available upon request.

## Strengths and limitations

The principal strengths of the COCOE Registry are its prospective, longitudinal design with follow-up to age 18; its use of validated, age-stratified, disease-specific instruments administered in both official languages; its embedding within an established multidisciplinary clinical pathway rather than a parallel research process; and the pre-specification of outcomes, analyses, missing data handling, and adherence metrics before any analysis is conducted.

Several limitations follow directly from the design and should temper interpretation of registry outputs. The registry is single-centre. Although COCOE serves a wide provincial and inter-provincial catchment, its case-mix reflects a tertiary referral pathway and is likely to be enriched for complex, syndromic, and reoperative presentations relative to the population of all children born with HD or ARM in Canada. Families living at a distance from Montréal, families without stable housing or telephone contact, and families whose preferred language is neither French nor English are plausibly under-represented at both enrolment and follow-up; the resulting bias would favour better-resourced families with better measured outcomes, which is the direction that most threatens the validity of continence and quality-of-life estimates. Registry outputs should therefore be read as describing a referred paediatric colorectal population rather than as national incidence or outcome estimates, and comparisons with ARM-Net and PCPLC data should be made with case-mix in mind [11,13]. The registry infrastructure, data dictionary, and REDCap instruments have been deliberately designed to be transferable to additional Canadian centres, and multicentre expansion through CanCORPS is the primary planned mitigation.

Combining HD and ARM within a single registry carries both operational and analytic risks. Operationally, the two cohorts share only part of the data model. Shared elements (demographics, social determinants of health, health service utilisation, PedsQL modules, PECI, PREOM-BMP, TRAQ, and the loss-to-follow-up definition) are captured on common forms, while disease-specific elements (Krickenbeck classification and the BCS for ARM; length of aganglionosis, HAEC ascertainment, and the BFS for HD) are captured on separate branching instruments, so that no clinician or abstractor is presented with a field that does not apply to the patient in front of them. Analytically, the two cohorts are never pooled for primary, survival, or multivariable analysis, and the events-per-variable threshold is assessed within each cohort separately. This is a conservative choice that may delay adjusted analyses in the smaller HD cohort. Pooling is confined to descriptive summaries of registry-level metrics such as enrollment, data completeness, and loss to follow-up, where disease group will always be reported as a stratifying variable. The main residual risk is therefore loss of statistical efficiency rather than confounding, and it is accepted in preference to a single combined model whose coefficients would be difficult to interpret clinically.

Finally, the primary outcome relies on instruments administered by proxy in younger children and by self-report from age 12, so the change of respondent within an individual’s follow-up may introduce measurement discontinuity. Respondent type will be included as a covariate in longitudinal models, and the pre-specified landmark estimates at ages 5 and 10 fall wholly within the proxy-report period, allowing an interpretation that is unaffected by the transition to self-report.

## Knowledge mobilisation and dissemination

This study uses an integrated Knowledge Translation (iKT) framework, with full inclusion of youth and parent research partners as key knowledge users [45]. Patient partners will review project aims and objectives, co-interpret results, continuously disseminate findings within patient and social networks, and plan further studies. Broader dissemination will occur locally through departmental presentations and nationally through CanCORPS (Canadian Consortium for Research in Pediatric Surgery), the Maternal Infant Child and Youth Research Network (MICYRN), the Alberta Surgery Strategic Clinical Network, and national professional associations. Results will be presented at international conferences and published in a minimum of two peer-reviewed manuscripts. Individual participants will not be identifiable in any publications.

## Protocol registration

This protocol has been registered with ClinicalTrials.gov. Registration number: NCT07603232. Date of registration: 2026-05-22.

## Data sharing statement

De-identified, aggregate data supporting findings from studies conducted using the COCOE Registry will be made available upon reasonable written request to the corresponding author (HW), subject to ethics approval from the requesting institution and execution of a data sharing agreement with the RI-MUHC. Individual-level participant data will not be shared publicly given the privacy obligations arising from the registry's consent framework and applicable Québec privacy legislation (Law 25). No data are available at the time of protocol publication.

As the registry has no fixed completion date, data sharing operates continuously rather than at a single terminal point. Specifically: (i) the full data dictionary, REDCap instrument definitions, and analysis code will be deposited in a public repository (Open Science Framework) at the time of first results publication and will carry a citable digital object identifier; (ii) the de-identified aggregate data underlying every figure and table in each registry publication will be provided as supporting information with that publication, in accordance with PLOS data policy; (iii) requests for de-identified individual-level data will be reviewed by a standing data access committee chaired by the registry Director against pre-specified criteria (scientific merit, ethics approval at the requesting institution, and an executed RI-MUHC data transfer agreement), with a target decision within 60 days and a publicly maintained log of approved requests; and (iv) should the registry cease operation, the de-identified dataset and its documentation will be transferred to a controlled-access repository under RI-MUHC custodianship so that it remains available for secondary research.

Individual-level data cannot be placed in an unrestricted public repository because the consent obtained from participants and the requirements of Québec Law 25 do not permit unrestricted redistribution of a longitudinal dataset of children with rare congenital conditions, for whom the combination of diagnosis, severity category, syndromic status, and follow-up dates carries a residual risk of re-identification. Requests may be directed to the corresponding author or to the RI-MUHC Research Ethics Office.

## Supporting information

S1 File SPIRIT 2025 checklist, adapted for observational registry protocols. Completed checklist mapping each SPIRIT 2025 item to the corresponding section of this protocol.
(DOCX)
