# Supplementary material for: Colorectal Centre of Excellence (COCOE) Registry: A protocol for a prospective longitudinal cohort of children with Hirschsprung's disease and anorectal malformations in Canada
Source: PLoS One. 2026 Aug 28;21(8):e0356813. doi: 10.1371/journal.pone.0356813 (PMC13524271; doi:10.1371/journal.pone.0356813)
Supplement: Supplementary file 1 [file pone.0356813.s001.docx]

**Participant timeline: Schedule of enrollment, interventions, and assessments.^a^**

|  | **COCOE Registry — SPIRIT 2025 Schedule of Enrolment and Assessments** | | | | | | | |
| --- | --- | --- | --- | --- | --- | --- | --- | --- |
|  | **Enrolment** | | **Follow-up (annual clinic visits)** | | | | | **Close-out / Transition** |
| **TIMEPOINT^b^** | ***-t_i_* to 0** | **0** | ***t_1_*** | ***t_2_*** | ***t_3_*** | ***t_4_*** | ***etc.*** | ***t_x_*** |
| **ENROLLMENT:** |  |  |  |  |  |  |  |  |
| Eligibility screen | X |  |  |  |  |  |  |  |
| Informed consent (parent/caregiver) | X |  |  |  |  |  |  |  |
| Age-appropriate assent (child) | X |  |  |  |  |  |  |  |
| Registry enrolment (REDCap ID assigned) | X |  |  |  |  |  |  |  |
| Re-consent at age 18 (adult transition) |  |  |  |  |  |  |  | X |
| **ASSESSMENTS: Baseline** |  |  |  |  |  |  |  |  |
| Demographics & social determinants of health | X | X |  |  |  |  |  |  |
| Diagnosis, phenotype & anomaly classification | X | X |  |  |  |  |  |  |
| Birth, perinatal & surgical history | X | X |  |  |  |  |  |  |
| Index surgical repair details |  | X |  |  |  |  |  |  |
| **ASSESSMENTS: Ongoing clinical** |  |  |  |  |  |  |  |  |
| Growth, examination & comorbidity update |  | X | X | X | X | X | X | X |
| Medications, hospitalisations & ED visits |  | X | X | X | X | X | X | X |
| Bowel management programme status |  | X | X | X | X | X | X | X |
| Subspecialist involvement |  | X | X | X | X | X | X | X |
| Surgical complications & reoperations |  | X | X | X | X | X | X | X |
| HAEC episodes (HD) |  | X | X | X | X | X | X | X |
| **PRIMARY OUTCOME:** |  |  |  |  |  |  |  |  |
| Time to fecal continence (ARM: BSC; HD: BFS ≥17) |  |  | X | X | X | X | X | X |
| **SECONDARY OUTCOMES:** |  |  |  |  |  |  |  |  |
| HAEC incidence & recurrence |  | X | X | X | X | X | X | X |
| Reoperation rate |  | X | X | X | X | X | X | X |
| Disease-specific QoL (HAQL) |  |  |  |  | X | X | X | X |
| Transition readiness (TRAQ) |  |  |  |  |  |  | X | X |
| Loss to follow-up rate |  | X | X | X | X | X | X | X |
| **PATIENT-REPORTED OUTCOMES / EXPERIENCE:** |  |  |  |  |  |  |  |  |
| PedsQL (general well-being & family impact) |  |  | X | X | X | X | X | X |
| Parent Experience of Child Illness (PECI) | X |  |  | X | X | X | X | X |
| PREOM-BMP (bowel management participants) |  |  | X | X | X | X | X | X |

^a^ Recommended content can be displayed using various schematic formats.

^b^ List target timepoints and acceptable time windows in this row (e.g., 30 ± 3 days).

^c^ BSC = Baylor Continence Scale; BFS = Bowel Function Score; ED = emergency department; HAEC = Hirschsprung-associated enterocolitis; HAQL = Hirschsprung’s and Anorectal Malformation Quality of Life; PECI = Parent Experience of Child Illness; PedsQL = Pediatric Quality of Life Inventory; PREOM-BMP = Patient-Reported Experience and Outcome Measures in Bowel Management Programmes; QoL = quality of life; TRAQ = Transition Readiness Assessment Questionnaire.

^d^ X indicates assessment performed at that timepoint. PREOM-BMP administered only to participants currently enrolled in a formal bowel management programme. Re-consent at age 18 sought at transition to adult care.

Citation: Chan A-W, Boutron I, Hopewell S, Moher D, Schulz KF, et al. SPIRIT 2025 statement: updated guideline for protocols of randomised trials. BMJ 2025;389:e081477. <https://dx.doi.org/10.1136/bmj-2024-081477>

© 2025 Chan A-W et al. This is an Open Access article distributed under the terms of the Creative Commons Attribution License (<https://creativecommons.org/licenses/by/4.0/>), which permits unrestricted use, distribution, and reproduction in any medium, provided the original work is properly cited.
