# Supplementary material for: Colorectal Centre of Excellence (COCOE) Registry: A protocol for a prospective longitudinal cohort of children with Hirschsprung's disease and anorectal malformations in Canada
Source: PLoS One. 2026 Aug 28;21(8):e0356813. doi: 10.1371/journal.pone.0356813 (PMC13524271; doi:10.1371/journal.pone.0356813)
Supplement: Supplementary file 2 [file pone.0356813.s002.docx]

Response to revision request

03 July 2026

Thank you for your revision request.

1) We have included the start date of the study in the cover letter (3^rd^ November 2025) and have outlined that the recruitment is still on-going with planned analyses to be conducted intermittently at several milestone time points throughout the study duration.
2) We have included the recruitment start date in the Methods section and have outlined that the study is currently on-going. We have highlighted that data analyses will be conducted at several milestone time points throughout the study as above and we anticipate results to be made available within the first two years.
3) We have uploaded the REB approval letter granting initial study approval, in addition to the final authorisation. The letter is bilingual in French and English.

Thank you again for considering our manuscript.
