# Supplementary material for: Colorectal Centre of Excellence (COCOE) Registry: A protocol for a prospective longitudinal cohort of children with Hirschsprung's disease and anorectal malformations in Canada
Source: PLoS One. 2026 Aug 28;21(8):e0356813. doi: 10.1371/journal.pone.0356813 (PMC13524271; doi:10.1371/journal.pone.0356813)
Supplement: Supplementary file 3 [file pone.0356813.s003.docx]

**Response to Reviewers**

Dear Editorial Team and Reviewers,

Thank you for the opportunity to revise our manuscript, and thank you to both reviewers for their careful and constructive assessment. We were pleased that both reviewers judged the rationale, protocol design, methodological detail, data availability plan, and language to be satisfactory, and we have used their specific questions to strengthen the protocol in the areas they identified.

Below we respond to each journal requirement and each reviewer comment in turn. Reviewer and editor comments are reproduced in italics; our responses follow. All changes are marked in the file labelled “Revised Manuscript with Track Changes”, and an unmarked version is supplied as “Manuscript”. Section names are given for each change; page and line numbers refer to the tracked-changes file.

The revision adds approximately 1,900 words, principally in four new or expanded passages: a new subsection on catchment and representativeness, a new subsection on data abstraction procedures and reliability, an expanded missing data plan, and a new Strengths and Limitations section. We believe these directly address the reviewers' questions without altering the design, outcomes, or analyses as originally registered.

# **Response to Journal Requirements**

## 1. **PLOS ONE style requirements and file naming**

## *Please ensure that your manuscript meets PLOS ONE's style requirements, including those for file naming.*

**Response:** The manuscript has been reformatted against the PLOS ONE main body and title/authors/affiliations templates. Headings now follow the PLOS hierarchy, the reference list follows PLOS numbered style with DOIs, tables are placed within the text with captions above, figure captions appear in the text after the paragraph of first citation, and files have been named according to the PLOS conventions (Manuscript, Revised Manuscript with Track Changes, Response to Reviewers, Fig1.tif, S1_File.docx).

**Change made:** Formatting applied throughout; no change to scientific content.

## 2. **Funding information**

## *Please remove any funding-related text from the manuscript and let us know how you would like to update your Funding Statement.*

**Response:** We apologise for the inconsistency. The Funding Statement section has been deleted in full from the manuscript, and the phrase “secured funding” has been removed from the Author Contributions statement so that no funding information remains anywhere in the manuscript file.

We would be grateful if the online submission form could be updated to the following Funding Statement, which is also reproduced in our cover letter:

“This registry is funded in part by funds received from the Montreal Children's Hospital Foundation (grant awarded to HW). The funders had no role in study design, data collection and analysis, decision to publish, or preparation of the manuscript.”

**Change made:** Funding Statement section deleted (previously located between Competing Interests and Author Contributions); Author Contributions amended.

## 3. **ORCID iD for the corresponding author**

## *PLOS requires an ORCID iD for the corresponding author in Editorial Manager.*

**Response:** The corresponding author's ORCID iD has been fetched and validated in Editorial Manager via ‘Update my Information’. Co-authors have additionally been asked to register and verify their own ORCID iDs within the submission system prior to final acceptance.

**Change made:** Administrative action in Editorial Manager; no change to the manuscript file.

## 4. **In-text citation of Figure 1**

## *Please ensure that you refer to Figure 1 in your text as, if accepted, production will need this reference to link the reader to the figure.*

**Response:** Thank you for catching this omission. Figure 1 is now explicitly cited in the text, and its caption has been placed in the manuscript immediately after the paragraph of first citation, in line with PLOS figure guidelines. The front-matter now also declares the number of figures.

**Change made:** Methods and Analysis – Study Design: sentence added citing Figure 1, followed by the caption “Fig 1. COCOE Registry workflow…”. Front matter: “Number of figures: 1” added. Please confirm that the caption wording matches the figure file as submitted and adjust if required.

## 5. **In-text citation of Table 3**

## *We note you have included a table to which you do not refer in the text of your manuscript. Please ensure that you refer to Table 3 in your text.*

**Response:** Table 3 is now cited in the text. In reviewing this we identified two related cross-referencing errors introduced during earlier drafting: the Outcome Measures section referred to “Table 1” where Table 2 was intended, and the Patient-reported outcomes section referred to “Table 2” where Table 3 was intended. All three tables are now cited correctly and in order of appearance, and the declared table count in the front matter has been corrected from two to three.

**Change made:** Outcome Measures (“Table 1” → “Table 2”); Patient-reported outcomes (“Table 2” → “Table 3”); front matter table count corrected.

## 6. **Supporting Information captions**

## *Please include captions for your Supporting Information files at the end of your manuscript, and update any in-text citations to match accordingly.*

**Response:** A Supporting Information section has been added at the end of the manuscript, immediately before the reference list, with a caption for the single supporting file. The in-text citation has been renamed from “Supplementary File 1” to “S1 File”, and the file itself has been renamed S1_File.docx to match.

**Change made:** New Supporting Information section added; in-text citation in the Missing Data section updated to “S1 File”.

## 7. **Reviewer-recommended citations**

## *If the reviewer comments include a recommendation to cite specific previously published works, please review and evaluate these publications…*

**Response:** Neither reviewer recommended specific additional citations. We have, however, added cross-references to the existing ARM-Net and PCPLC references [11,13] and to the Canadian incidence references [1,3,4] within the new text, since these are the benchmarks against which representativeness will be assessed. No new references have been added to the reference list.

# **Response to Reviewer #1**

# *The authors have presented a protocol for prospective longitudinal recording of results. Such information is much needed. It is quite an ambitious protocol, and it will be interesting to see how good the adherence to the protocol will be.*

**Response:** We thank Reviewer #1 for this supportive assessment and for identifying protocol adherence as the key practical risk. We agree entirely: a longitudinal registry running to age 18 stands or falls on adherence, and a protocol that does not commit in advance to measuring and publishing its own adherence gives readers no way to judge the completeness of the data behind its results.

We have therefore made adherence an explicitly monitored and reportable registry metric rather than an implicit assumption. Four indicators are now pre-specified: the proportion of eligible patients approached, the proportion consented, the proportion of scheduled assessments completed within their pre-specified age window, and instrument-level completion rates. These are reported quarterly to the registry Director, with a pre-specified action threshold (assessment-window adherence below 70% across two consecutive quarters) that triggers review of clinic workflow and, if needed, a protocol amendment. Crucially, we have committed to publishing these adherence metrics alongside every outcome analysis, so that the question the reviewer raises can be answered from the published record rather than taken on trust.

We have also added a linked commitment in the Missing Data section that no outcome analysis will be reported without its accompanying completeness figures, and a threshold above which model-based inference will not be attempted.

**Change made:** Data Collection – new subsection “Data Abstraction Procedures and Reliability”, third paragraph (protocol adherence metrics and action thresholds); Monitoring and Data Quality Oversight (annual data quality report); Missing Data (completeness reporting requirement).

# **Response to Reviewer #2**

## **General assessment and generalisability**

## *The manuscript presents a well-structured, single-centre prospective registry protocol… the registry's single-centre nature and regional catchment may limit generalisability and should be more clearly discussed.*

**Response:** We accept the criticism. The original manuscript had no limitations section at all, and the generalisability constraint was left implicit. We have addressed this in two places.

First, we have added a new Methods subsection, “Catchment and Representativeness”, which describes the referral catchment concretely and, more importantly, commits to quantifying representativeness rather than asserting it. At each annual analysis the age, sex, diagnosis and severity distribution of enrolled participants will be compared against (i) expected provincial case counts derived by applying published Canadian incidence estimates for HD and ARM to Québec live-birth denominators, and (ii) the published case-mix of ARM-Net and PCPLC. We have also added prospective aggregate recording of patients who are approached but decline, by age band, diagnosis, and referral region, so that a non-participation comparison can be reported.

Second, we have added a new “Strengths and Limitations” section near the end of the manuscript. This states plainly that the case-mix reflects a tertiary referral pathway and is likely enriched for complex, syndromic, and reoperative presentations; that families at a distance, without stable contact details, or whose preferred language is neither French nor English are plausibly under-represented at both enrolment and follow-up; and that the resulting bias would run toward better-resourced families with better measured outcomes — the direction that most threatens the validity of continence and quality-of-life estimates. We now state explicitly that registry outputs should be read as describing a referred paediatric colorectal population rather than as national estimates, and we identify multicentre expansion through CanCORPS as the primary planned mitigation, which the registry's transferable data dictionary and REDCap instruments are designed to support.

**Change made:** New subsection “Catchment and Representativeness” (Study Population and Setting); new section “Strengths and Limitations” (before Knowledge Mobilisation and Dissemination).

## **Question 1. How will the team ensure consistency in clinical data abstraction from multidisciplinary forms?**

**Response:** This was under-specified in the original submission and we thank the reviewer for pressing on it. We have added a new Methods subsection, “Data Abstraction Procedures and Reliability”, describing four layers of control.

Documentation: the registry operates under a written data dictionary and standard operating procedure that specify, for every REDCap field, the permitted values, the source document of record, and the rule applied when sources disagree. Operative and histopathology reports take precedence over clinic correspondence for surgical and diagnostic variables; the multidisciplinary assessment form takes precedence for functional and psychosocial variables. This last point matters specifically for multidisciplinary forms, where the same variable may be recorded by more than one discipline.

Training and certification: all abstractors complete a standardised training package and must reach at least 90% field-level agreement with the Study Coordinator across five benchmark charts before abstracting independently.

Instrument design: REDCap forms use closed-response fields, branching logic, and range and cross-field validation, so free-text entry is reserved for variables that cannot be pre-coded.

Ongoing measurement: a random 10% sample of records is independently re-abstracted each quarter by a second trained abstractor blinded to the original entry. Agreement is quantified using Cohen's kappa for categorical variables and the intraclass correlation coefficient for continuous variables, against a pre-specified threshold of κ ≥ 0.80 or ICC ≥ 0.80. Fields falling below threshold trigger review of the field definition, retraining, and re-abstraction of affected records. Reliability statistics will be reported in the first descriptive registry manuscript and at each annual audit thereafter.

**Change made:** Data Collection – new subsection “Data Abstraction Procedures and Reliability”; Monitoring and Data Quality Oversight expanded to include reliability statistics in the annual data quality report.

## **Question 2. How will the registry data be shared upon study completion?**

**Response:** The original Data Sharing Statement was written for a study with a defined endpoint, which this registry does not have, and it was correspondingly vague about mechanism. We have rewritten it to describe sharing as a continuous process with four concrete components.

(i) The full data dictionary, REDCap instrument definitions, and analysis code will be deposited in a public repository (Open Science Framework) at the time of first results publication, with a citable DOI. (ii) The de-identified aggregate data underlying every figure and table in each registry publication will be provided as supporting information with that publication, in accordance with PLOS data policy. (iii) Requests for de-identified individual-level data will be reviewed by a standing data access committee chaired by the registry Director against pre-specified criteria — scientific merit, ethics approval at the requesting institution, and an executed RI-MUHC data transfer agreement — with a target decision within 60 days and a publicly maintained log of approved requests. (iv) Should the registry cease operation, the de-identified dataset and its documentation will be transferred to a controlled-access repository under RI-MUHC custodianship so that it remains available for secondary research.

We have also stated the reason for controlled rather than open access explicitly, since we think readers are owed the reasoning: the consent obtained and the requirements of Québec Law 25 do not permit unrestricted redistribution of a longitudinal dataset of children with rare congenital conditions, for whom the combination of diagnosis, severity category, syndromic status, and follow-up dates carries a residual re-identification risk.

**Change made:** Data Sharing Statement expanded (two new paragraphs).

## **Question 3. What statistical strategies will be used to address missing PRO data and incomplete follow-up, especially given the long follow-up window to age 18?**

**Response:** We agree this is the central analytic threat over a follow-up window of this length, and the original missing data plan was too generic. The Missing Data section now sets out a pre-specified hierarchy matched to the level at which data are missing.

Item level: partially completed questionnaires are prorated according to each developer's published scoring rule; scale scores are set to missing where fewer than the minimum required items are available.

Visit level: longitudinal PRO analyses use linear mixed-effects models and generalised estimating equations, which retain participants with incomplete visit series and give valid inference under a missing-at-random assumption, rather than deleting incomplete records.

Covariate level: multiple imputation by chained equations is used for multivariable models, with the imputation model including the analysis variables, the outcome, and auxiliary variables predictive of non-response — age band, distance from COCOE, deprivation quintile, language of administration, and number of previously missed appointments.

Informative dropout: because loss to follow-up and PRO non-response may share causes, inverse probability of censoring weights estimated from these same predictors are applied as a sensitivity analysis to the survival and longitudinal models.

Departures from MAR: robustness is assessed using pattern-mixture models in which imputed PRO scores are delta-adjusted by 0.2, 0.5, and 0.8 standard deviations in the unfavourable direction. If conclusions do not survive a 0.5 SD adjustment we will say so.

Finally, we have added two reporting commitments. Missingness will be tabulated by instrument, visit, and disease cohort in every registry publication; and where missingness for a given instrument at a given assessment point exceeds 40%, model-based inference will not be attempted and results will be reported descriptively.

**Change made:** Missing Data section: two new paragraphs added following the existing MICE and MNAR text.

## **Question 4. Although analyses will be disease-specific, does combining HD and ARM in a single registry risk operational or analytic heterogeneity?**

**Response:** Yes, and we think it is right to name the risk rather than defend the decision. We have added a paragraph to the new Strengths and Limitations section that separates the two forms of heterogeneity and states what is done about each, and what residual risk is accepted.

Operationally, the two cohorts share only part of the data model. Shared elements — demographics, social determinants of health, health service utilisation, the PedsQL modules, PECI, PREOM-BMP, TRAQ, and the loss-to-follow-up definition — are captured on common forms. Disease-specific elements — Krickenbeck classification and the BCS for ARM; length of aganglionosis, HAEC ascertainment, and the BFS for HD — are captured on separate branching instruments, so no clinician or abstractor is presented with a field that does not apply to the patient in front of them. This is the practical mechanism by which the shared infrastructure avoids becoming a source of abstraction error.

Analytically, the cohorts are never pooled for primary, survival, or multivariable analysis, and the events-per-variable threshold is now assessed within each cohort separately rather than across the registry as a whole. We acknowledge in the manuscript that this is conservative and may delay adjusted analyses in the smaller HD cohort. Pooling is confined to descriptive summaries of registry-level metrics — enrolment, completeness, loss to follow-up — where disease group is always reported as a stratifying variable.

We state the resulting trade-off explicitly: the main residual risk is loss of statistical efficiency rather than confounding, and we accept it in preference to a combined model whose coefficients would be difficult to interpret clinically.

**Change made:** Strengths and Limitations, third paragraph; the existing rationale in Study Population and Setting is retained and now cross-referenced by the new material.

## **Question 5. How representative is the COCOE patient population of the broader Canadian paediatric colorectal population?**

**Response:** We cannot yet answer this quantitatively, and we have been careful in the revision not to imply otherwise. What we can do at the protocol stage is commit in advance to how the question will be answered, and to reporting the answer whatever it shows.

The new “Catchment and Representativeness” subsection describes the referral base — the Montreal Children's Hospital is one of two paediatric tertiary centres in Québec and receives referrals from across the province, including from Nunavik and the Cree territory of James Bay, together with selected complex interprovincial referrals — and then sets out the benchmarking plan: annual comparison of the enrolled population's age, sex, diagnosis and severity distribution against expected provincial case counts derived from published Canadian incidence estimates applied to Québec live-birth denominators, and against the published case-mix of ARM-Net and PCPLC. Aggregate, non-identifying data on those who decline enrolment will support a non-participation comparison.

We also commit to reporting divergence explicitly — under-representation of remote communities or of the most severe Krickenbeck categories is named as an example — and to carrying that divergence into the interpretation of external validity. The Strengths and Limitations section states that, until multicentre data are available, registry outputs describe a referred paediatric colorectal population and should not be read as national incidence or outcome estimates.

**Change made:** New subsection “Catchment and Representativeness”; Strengths and Limitations, second paragraph.

# **Additional Changes Made at Revision**

In reviewing the manuscript we identified and corrected the following, none of which alters the design or analysis:

- Objectives: the numbered list was mis-numbered and ran 1), 2), 3) across five objectives. It now runs 1) to 5) with no change to content.
- Introduction: an orphaned superscript citation marker (“8”) after “Colorectal Centre of Excellence (COCOE)”, a residue of an earlier citation style, has been deleted.
- Study Timeline and Milestones: “opened to enrolment on the 3rd November 2025 and are currently on-going” corrected to “opened to enrolment on 3 November 2025 and enrolment is ongoing”.
- Statistical Analysis, Objective 1: a missing full stop before “Where competing risks are present” has been inserted.
- Statistical Analysis, Objective 2: “selected by Akaike Information” corrected to “selected by the Akaike Information Criterion”.
- Survey Administration and Burden Mitigation: “Surveys available in English and French” corrected to “Surveys are available in English and French”.
- Front matter: word count and table count updated; figure count added.

We hope the revised manuscript is now suitable for publication in PLOS ONE, and we thank the editor and both reviewers again for comments that have materially improved the protocol. We would be glad to make any further changes required.

Yours sincerely,

**Hussein Wissanji, MD, MPH, FRCS**

On behalf of all co-authors

Harvey E. Beardmore Division of Pediatric Surgery, Montreal Children's Hospital, McGill University Health Centre

hussein.wissanji@mcgill.ca
